# Supplementary material for: Developing a programme theory of a complex, home-based rehabilitation intervention for recovery after an episode of delirium
Source: PLoS One. 2026 Mar 31;21(3):e0342149. doi: 10.1371/journal.pone.0342149 (PMC13038012; doi:10.1371/journal.pone.0342149)
Supplement: S1 File — Document containing the repository details and permanent link to the anonymised minimal dataset underlying the results reported in this study. (DOCX) [file pone.0342149.s001.docx]

[Anonymised qualitative interview excerpts underpinning Phase 2 of the RecoverED programme theory development process](https://ore.exeter.ac.uk/articles/dataset/Anonymised_qualitative_interview_excerpts_underpinning_Phase_2_of_the_RecoverED_programme_theory_development_process/31161238)

Authors (15)

Shruti Raghuraman, Sarah Morgan-Trimmer, Robert Anderson, Victoria Goodwin, Linda Clare, Ellen Richards, Aseel Mahmoud, Alison Bingham, Elizabeth Goodwin, Rowan Harwood, Annemarie Hawton, Sarah Richardson, Jinpil Um, Obioha Ukoumunne, Louise Allan

Creation date

2026-01-27

Size

32.52 KB

Identifier <https://doi.org/10.24378/exe.31161238>
