# Supplementary material for: Developing a programme theory of a complex, home-based rehabilitation intervention for recovery after an episode of delirium
Source: PLoS One. 2026 Mar 31;21(3):e0342149. doi: 10.1371/journal.pone.0342149 (PMC13038012; doi:10.1371/journal.pone.0342149)
Supplement: Attachment — Submitted filename: Response to Reviewers.docx [file pone.0342149.s003.docx]

**PONE-D-25-52519**

Developing a programme theory of a complex, home-based rehabilitation intervention for recovery after an episode of delirium

**Response to Reviewers**

| **Source** | **Required Action** | **Response** |
| --- | --- | --- |
| PLOS Editor | Please ensure that your manuscript meets PLOS ONE's style requirements, including those for file naming. |  |
| PLOS Editor | Please describe in your methods section how capacity to provide consent was determined for the participants in this study. Please also state whether your ethics committee or IRB approved this consent procedure. If you did not assess capacity to consent please briefly outline why this was not necessary in this case. | The current paper reports only secondary synthesis of previously published work. Each underlying study had its own IRB/ethics approval, consent procedures, and data-sharing statements, and no new ethics review, capacity assessment, or data collection was required for this paper. Where relevant, citations have been included within this paper, and will be updated as more DOIs become available. |
| PLOS Editor | In the ethics statement in the Methods, you have specified that verbal consent was obtained. Please provide additional details regarding how this consent was documented and witnessed, and state whether this was approved by the IRB | The current paper reports only secondary synthesis of previously published work. Each underlying study had its own IRB/ethics approval, consent procedures, and data-sharing statements (as cited); and no new ethics review, capacity assessment, or data collection was required for this paper. A paragraph detailing this has been added via tracked changes to the Methods section, 'Ethics statement'. (p.6). |
| PLOS Editor | Your ethics statement should only appear in the Methods section of your manuscript. If your ethics statement is written in any section besides the Methods, please move it to the Methods section and delete it from any other section. Please ensure that your ethics statement is included in your manuscript, as the ethics statement entered into the online submission form will not be published alongside your manuscript. | Thank you. This is now done (p.6) |
| PLOS Editor | We noted in your submission details that a portion of your manuscript may have been presented or published elsewhere. Figure 2. Preliminary programme theory Adapted from O’Rourke et al., 2020 (PMID: 32734773), representing Stage 1 of programme theory development. This initial framework was subsequently refined and expanded through the multi-stage process reported in the present study.   Please clarify whether this [conference proceeding or publication] was peer-reviewed and formally published. If this work was previously peer-reviewed and published, in the cover letter please provide the reason that this work does not constitute dual publication and should be included in the current manuscript | We wish to clarify that Figure 2 (“Preliminary programme theory”) is adapted from O’Rourke et al. (2020; PMID: 32734773), which represents Stage 1 of the multi-stage programme theory development process. That paper reported the findings of the initial rapid realist review, which formed the foundation for the subsequent stages described in the present manuscript.  The current manuscript does not duplicate or reproduce the published material but builds upon it to describe the iterative refinement and synthesis of the programme theory across multiple stages, integrating findings from later qualitative and feasibility phases. Therefore, this submission does not constitute dual publication but provides a higher-level synthesis of the full theory-development process. |
| PLOS Editor | We note that you have indicated that there are restrictions to data sharing for this study. For studies involving human research participant data or other sensitive data, we encourage authors to share de-identified or anonymized data. However, when data cannot be publicly shared for ethical reasons, we allow authors to make their data sets available upon request.  Please update your Data Availability statement in the submission form accordingly. | This has been clarified under 'Data availability' -  No new data were generated or analysed as part of this publication. The datasets underpinning the individual studies are already, or will be accessible through their respective publications. Re-depositing these datasets would duplicate existing records and may contravene the original data-sharing agreements and ethics approvals. |
| PLOS Editor | In the online submission form, you indicated that data are available upon reasonable request.   All PLOS journals now require all data underlying the findings described in their manuscript to be freely available to other researchers, either 1. In a public repository, 2. Within the manuscript itself, or 3. Uploaded as supplementary information. This policy applies to all data except where public deposition would breach compliance with the protocol approved by your research ethics board. If your data cannot be made publicly available for ethical or legal reasons (e.g., public availability would compromise patient privacy), please explain your reasons on resubmission and your exemption request will be escalated for approval. | This has been clarified under 'Data availability' -  No new data were generated or analysed as part of this publication. The datasets underpinning the individual studies are already, or will be accessible through their respective publications. Re-depositing these datasets would duplicate existing records and may contravene the original data-sharing agreements and ethics approvals. |
| PLOS Editor | We note that the grant information you provided in the ‘Funding Information’ and ‘Financial Disclosure’ sections do not match.  When you resubmit, please ensure that you provide the correct grant numbers for the awards you received for your study in the ‘Funding Information’ section. | The 'Funding Information' section in the submission has been updated to include the start-up grant receieved by Prof Louise Allan. |
| PLOS Editor | Add statement on role of funders or include required sentence and place corrected version in cover letter. | This has been done (p.32) |
| PLOS Editor | Add explicit references in text to Tables 1–7. | This has been added under tracked changes. |
| PLOS Editor | Review reviewer-recommended citations and add only if relevant. | Not relevant as reviewers have not suggested adding any references. |
| PLOS Editor | Check references remove or justify retracted references and note changes in rebuttal. | Not relevant. |
| Reviewer #1 | Phase 4: the research team consisted of a wide MDT; however, the voice of nursing was missing. Can you please clarify this? | We thank the reviewer for this helpful observation. The core University of Exeter research team did not include a member with nursing expertise. We agree that this represents a gap in professional representation, and we have now acknowledged this in the revised manuscript (Phase 4, Methods section). |
| Reviewer #1 | Page 9: Who were the HCP participants? Suggest making this clearer. I think it is important to show that you captured all the voices of the MDT, including nursing. If you did not capture the voices of nursing staff, then I think this needs to be discussed as a limitation. | We thank the reviewer for this helpful observation. This comment refers to the embedded process evaluation conducted within the feasibility trial. The RecoverED intervention was designed as a community-based, face-to-face rehabilitation programme delivered by a multidisciplinary team consisting of occupational therapists, physiotherapists, and rehabilitation support workers. The process evaluation therefore included the views of those who planned and delivered the intervention; reflecting the composition of the actual facilitation team. Nursing professionals were not part of the intervention delivery team, and consequently their perspectives were not captured within this process evaluation. To address the reviewer's comment, we have now added a sentence on page 9 to elaborate on the composition of the healthcare professional (HCP) participants for clarity (p.10) |
| Reviewer #1 | I wonder if there were any negative attitudes towards delirium that need to be challenged/ addressed in order to make the program success? Did the education challenge attitudes? | We thank the reviewer for this thoughtful comment. No negative or stigmatising attitudes towards delirium were identified in this study. Participants generally demonstrated awareness of the challenges associated with delirium and recognised the importance of person-centred, multidisciplinary approaches. The educational component of the RecoverED intervention was viewed as reinforcing and consolidating existing understanding rather than challenging negative attitudes. |
| Reviewer #1 | Discuss how programme supports participants without carers. | Thank you for raising this important point. We identified the absence of formal or informal carers as a limitation within our inclusion criteria, as this meant that individuals without a carer were not represented, despite potentially having similar rehabilitation needs. We have added a line in the Discussion section to acknowledge this, and highlight the need for future adaptations of the RecoverED intervention to address this group (p.29 - 30). |
| Reviewer #1 | Correct bracket formatting error in context mapping sentence. | Thank you, this error has been corrected. |
| Reviewer #1 | Fix incorrect reference formatting. | Thank you, this error has been corrected. |
| Reviewer #1 | Add citation for process evaluation published elsewhere. | The process evaluation manuscript is currently under review following submission of its first revision. We will update the citation with the DOI and full reference details once it is accepted and published. |
| Reviewer #2 | I note that data will be made available upon request, rather than included in the manuscript. | The Data Availability Statement has been updated to reflect the following -  No new data were generated or analysed as part of this publication. The datasets underpinning the individual studies are already, or will be accessible through their respective publications. Re-depositing these datasets would duplicate existing records and may contravene the original data-sharing agreements and ethics approvals. |
